# Uncovering the Diagnostic Challenge of Myelin Oligodendrocyte Glycoprotein Antibody-Associated Disease: A Case Study of Acute Bilateral Vision Loss

**DOI:** 10.7759/cureus.60612

**Published:** 2024-05-19

**Authors:** Anandu M Anto, Sai Vishnu Vardhan Allu, Samrachana Acharya, Trupti Vakde, Eghosa Omoregi, Udesh Pandey

**Affiliations:** 1 Medicine, BronxCare Health System, New York, USA; 2 Internal Medicine, BronxCare Health System, New York, USA; 3 Pulmonary and Crticial Care, BronxCare Health System, New York, USA; 4 Neurology, BronxCare Health System, New York, USA

**Keywords:** neuro-opthalmology, multiple sclerosis behavior, neuro-critical care, bilateral optic neuritis, inflammatory and demyelinating disease, nmo spectrum disorder, myelin-oligodendrocyte glycoprotein antibody disease

## Abstract

We discuss a perplexing case of a 51-year-old female with a history of asthma and morbid obesity, presenting with acute bilateral vision loss of unknown etiology. The patient's clinical course was marked by a constellation of symptoms, including blurry vision, eyeball pain, photophobia, headache, nausea, and dizziness, prompting a multidisciplinary approach for diagnostic evaluation. Despite a comprehensive workup and a temporal artery biopsy ruling out large vessel arteritis, the etiology of vision loss remained elusive until myelin oligodendrocyte glycoprotein (MOG) antibody testing returned positive, implicating myelin oligodendrocyte glycoprotein antibody-associated disease (MOGAD). High-dose corticosteroid therapy was initiated. However, the patient had worsening visual symptoms and was started on plasmapheresis and subsequent administration of Rituximab to prevent relapses, along with a long-term steroid taper regimen. This case underscores the diagnostic challenge of optic neuritis, particularly in MOGAD. It emphasizes the importance of a thorough evaluation and multidisciplinary collaboration.

## Introduction

Optic neuritis, characterized by optic nerve inflammation, can be idiopathic or associated with various systemic conditions, including autoimmune diseases. Myelin oligodendrocyte glycoprotein antibody-associated disease (MOGAD) represents a spectrum of autoimmune disorders involving the central nervous system, including optic neuritis [1,2]. The prevalence of MOGAD is 1.3-2.5/100,000, and the annual incidence is 3.4-4.8 per million [3]. A disease of autoimmune etiology with a good response to steroids, however, tends to relapse after stopping corticosteroids, necessitating suppressive therapies with Rituximab [4,5]. The key differentials in patients with optic neuritis are multiple sclerosis (MS), neuromyelitis optica (NMO), and MOGAD. The rarity of MOGAD and its diverse clinical manifestations pose diagnostic dilemmas, necessitating a high index of suspicion and a comprehensive diagnostic approach [6].

## Case presentation

A 51-year-old female with a history of asthma was referred to the Emergency Department (ED) by her ophthalmologist due to disc swelling and a rapid decrease in vision. The patient reported the onset of blurry vision, eye pain, photophobia, headache, nausea, and dizziness starting three days before the presentation. A fundus examination revealed bilateral blurred disc margins. The patient underwent a CT angiogram of the brain in the emergency room (ER), which was unremarkable. Visual acuity worsened on Day 3 of admission, which prompted the urgent initiation of methylprednisolone. Laboratory investigations revealed a normal hemogram, coagulation profile, renal function, and hepatic function tests (Tables 1, 2, 3).

**Table 1:** Laboratory results CBA, cell-based assay; ESR, erythrocyte sedimentation rate; ANA, anti-nuclear antibody; AQP4 Ab, aquaporin 4 antibody, NMO, neuromyelitis Optical; Ab, antibody

Test name	Result	Reference range
ESR	60 mm/hr	<30 mm/hr
Lyme test	Negative	<0.90
Immunoglobulin G level, serum	1310	600-1640 mg/dL
ANA	Negative	
Myeloperoxidase	Negative	<1.0
Proteinase-3 Ab	Negative	<1.0
SS-A Ab	Negative	<1.0
SS-B Ab	Negative	<1.0
Rheumatoid factor, serum	<10 U/mL	≤14 IU/mL
Angiotensin converting enzyme	22 U/L	9-67 U/L
AQP4 Ab NMO-Ig G ELISA	Negative	
MOG AB CBA, serum	Positive	Negative
MOG AB titer, serum	0.152778	<1:10 titer

**Table 2:** CSF study CSF, cerebrospinal fluid study

Parameter	Result
CSF color	Colorless
CSF appearance	Clear
WBC count	3
RBC count	6
Total cells	0
Glucose	58 (40-70mg/dL)
Protein	25 (15-45 mg/dL)

**Table 3:** MS panel, CSF CSF, cerebrospinal fluid study; MS, multiple sclerosis

MS panel, CSF	
Oligoclonal bands	Absent
Albumin	14.7 (8-42 mg/dL)

**Figure 1:**
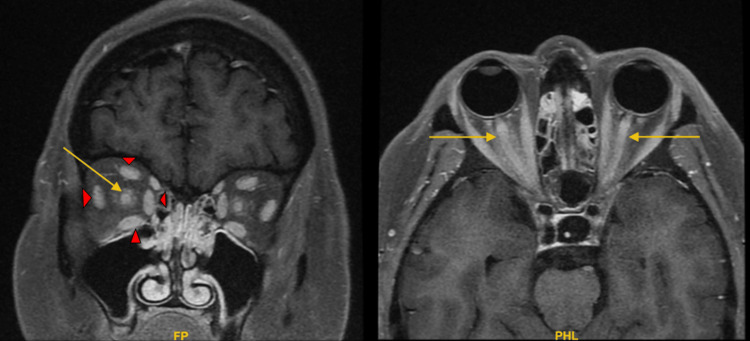
Normal enhancement of the extraocular muscles (red arrowheads) and the abnormal diffuse enhancement of the bilateral optic nerves involving approximately 27 mm of intraorbital segment, right more than left (yellow arrows)

The patient underwent an MRI brain, MR venogram, and MRI orbit on day 3. MRI orbit revealed the abnormal diffuse enhancement of the bilateral optic nerves involving approximately 27 mm of the intraorbital segment, right more than left, which led to suspicion of NMO vs MS (Figure 1). The patient was evaluated by neurology and neuro-ophthalmology, and they recommended a lumbar puncture. Lumbar puncture was done on day 3, and it did not reveal any evidence of any infection nor any evidence of MS (negative oligoclonal bands), with an opening pressure of 14 cm H_2_0. The differential at this point was NMO. However, AQP4 antibodies were negative, prompting evaluation for MOGADS. The MOG antibody test returned positive, as noted in the results table. MRI spine revealed spondylotic changes and neural foraminal narrowing with concern for L5 nerve impingement at L5-S1. Temporal artery biopsy was negative for giant cell arteritis. Bilateral optic neuritis, noted in MRI orbits, a hallmark of MOGAD, was critical in guiding the diagnosis and the treatment plan.

Since the patient’s visual acuity was not improving despite a high dose of methylprednisolone (Methylprednisolone 1g bolus on the first day, followed by 250mg every 6 hours for 2 days), the patient was initiated on plasmapheresis on day 6 of admission. The patient underwent 5 sessions of plasmapheresis on alternate days, followed by the administration of Rituximab. Rituximab was started to prevent relapses. After a comprehensive assessment, the patient was deemed medically stable for discharge. Post-discharge, she continued follow-up with neurology and ophthalmology. She commenced a prednisone taper and continued Rituximab infusions as outpatient care. The patient felt better, and her vision improved to near normal.

## Discussion

Historically, patients who manifested symptoms of optic neuritis and acute transverse myelitis were initially diagnosed with NMO via the presence of immunoglobulin G (IgG) against the water channel aquaporin-4 (AQP4 IgG) expressed by astrocytes. However, it was noted that 10-25% of patients who were suspected to have NMO were negative for AQP4 antibodies [7]. This led to the search for other antibodies against components of the CNS and finally led to the identification of antibodies against MOG. MOG is one of several proteins produced by oligodendrocytes. It is an essential part of the oligodendrocyte surface membrane along with myelin basic protein (MBP), proteolipid protein (PLP), and myelin-associated glycoprotein (MAG) [8]. MOG antibodies were identified in 21% of AQP4 IgG seronegative patients with optic neuritis [9].

MOGAD is an autoimmune demyelinating disorder of the central nervous system defined by antibodies against MOG, a component critical for the integrity of the myelin sheath [7]. 

Clinical presentation and prognosis in MOG-antibody disease: a UK study, Jurynczyk et al., identified that 55% of the cases had presented with optic neuritis, 24% were bilateral, 14% were longitudinal and extensive, 4% had short segment involvement, 9% had simultaneous optic neuritis and transverse myelitis, and 18% had ADEM (acute disseminated encephalomyelitis) or ADEM-like presentation [4]. 34% of MOGAD patients had brainstem or cerebellar involvement, and 63% were symptomatic. Ataxia (45%) and diplopia (26%) were common manifestations [10]. It was also noted that patients within the age group of 20-45 years most often presented with unilateral optic neuritis. In contrast, patients above 45 years of age presented with bilateral optic neuritis [4]. Optic neuritis is characterized by acute, often painful, vision loss [11]. In our case, the patient was 51 years old and presented with a painful blurring of vision and was noted to have optic neuritis bilaterally. The differential diagnoses for MOGAD optic neuritis encompass a variety of conditions capable of causing optic nerve inflammation, including MS, NMOSD (NMO spectrum disorder), infectious etiologies (e.g., viral and bacterial), and other autoimmune processes (e.g., sarcoidosis) [12]. NMOSD, associated with AQP4 antibodies, typically presents with more severe optic neuritis and spinal cord involvement but does not involve MOG antibodies. Patients with MOGAD optic neuritis may exhibit visual impairment, ocular pain, especially with movement, and dyschromatopsia. Uhthoff's phenomenon, a transient worsening of symptoms with increased body temperature, may also be observed. Key distinguishing features of MOGAD include a typically more pronounced response to corticosteroids and a higher propensity for relapse compared to MS optic neuritis (Table 4) [5,13].

**Table 4:** Features differentiating MS, NMO, and MOGAD NMO, neuromyelitis optics; MOGAD, myelin oligodendrocyte glycoprotein antibody-associated disease; MS, multiple sclerosis

Feature	MS	NMO	MOGAD
Optic neuritis [12,14-16]	Unilateral, segmental optic nerve involvement	Unilateral or bilateral, extensive optic nerve involvement	Unilateral or bilateral with extensive optic nerve involvement
Transverse myelitis [17]	Segmental and rarely involves conus medullaris	Extensive and rarely involves conus medullaris	Segmental or extensive and commonly involves conus medullaris
Clinical course [18-20]	90% relapsing-remitting	90% relapsing vs 10% monophasic	40-50% monophasic vs relapsing in 50-55% cases
CSF [21]	Normal to mild lymphocytic pleocytosis, common to see oligoclonal bands	Elevated neutrophils and eosinophils, uncommon oligoclonal bands	Markedly elevated lymphocytic pleocytosis, uncommon oligoclonal bands
Serology [22]	Clinical diagnostic criteria and presence of oligoclonal bands	Aquaporin-4 antibody positive	MOG antibody positive

Diagnostic evaluation includes MRI, which may demonstrate optic nerve enhancement with gadolinium contrast indicative of inflammation. Although brain and spinal cord lesions can be present, their distribution often differs from that observed in MS. In the study by Mealy et al. evaluating longitudinal extensive optic neuritis as an MRI biomarker to distinguish NMO from MS, they noted that most of the NMO lesions were longitudinally extensive, measuring at least 17.6 mm in length. Optic nerve lesion length of at least 17.6 mm was noted to have a sensitivity of 80.8% and a positive likelihood ratio of 3.50 [23]. Meanwhile, MS lesions are more commonly focal in the anterior part of either one of the optic nerve segments. In our patient, long segment enhancement of the bilateral optic nerves (approx. 27 mm), intraorbital segments, was noted compared to the prechiasmatic optic nerves and optic chiasm, suggesting a high sensitivity and likelihood of the diagnosis being NMO. The right optic nerve appeared slightly larger compared to the left optic nerve. Laboratory investigations are crucial in differentiating MOGAD from other demyelinating diseases. The AQP4 antibody was negative in our patient, and the MOG-IgG antibody was positive. A positive serum MOG-IgG antibody test is pivotal for the diagnosis, setting MOGAD apart from MS and NMOSD, characterized by oligoclonal bands in cerebrospinal fluid (CSF) and AQP4 antibodies, respectively. CSF analysis in MOGAD may reveal pleocytosis but typically lacks the oligoclonal bands seen in MS.

Managing acute MOGAD optic neuritis primarily involves high-dose intravenous corticosteroids, such as methylprednisolone, to reduce inflammation and hasten recovery [24]. For cases unresponsive to steroids or those with recurrent episodes, plasma exchange (PLEX) or intravenous immunoglobulin (IVIG) may be employed [2]. Considering the relapsing nature of MOGAD, long-term immunosuppression with agents like rituximab, mycophenolate mofetil, or azathioprine may be necessary to mitigate the risk of future relapses [25].

## Conclusions

In conclusion, the case of MOGAD's disease in this 51-year-old woman underscores the importance of prompt diagnosis and appropriate treatment. The clinical presentation of MOGAD's disease can be variable and nonspecific, often leading to diagnostic challenges. However, early recognition and accurate diagnosis with extensive and broad workup are crucial to initiate timely treatment and prevent potential complications such as cardiovascular and thrombotic events. Moreover, managing MOGAD's disease involves a multidisciplinary approach, including immunosuppressive therapy and supportive care tailored to individual patient needs. Continued research efforts are warranted to further elucidate this rare disorder's pathophysiology and optimize therapeutic strategies, improving outcomes for patients with MOGAD's disease.
